# Blood-patch pleurodesis for pneumothorax in lung fibrosis due to progressive systemic sclerosis: A case report

**DOI:** 10.3892/etm.2014.1993

**Published:** 2014-09-29

**Authors:** KATSUNORI KAGOHASHI, GEN OHARA, TOSHIHIRO SHIOZAWA, TOMOHIRO TAMURA, KUNIHIKO MIYAZAKI, KOICHI KURISHIMA, HIROAKI SATOH, NOBUYUKI HIZAWA

**Affiliations:** 1 Division of Respiratory Medicine, Mito Medical Center, University of Tsukuba, Mito, Ibaraki 310-0015, Japan; 2 Division of Respiratory Medicine, Faculty of Medicine, University of Tsukuba, Mito, Ibaraki 310-0015, Japan

**Keywords:** blood-patch pleurodesis, pneumothorax, lung fibrosis, progressive systemic sclerosis

## Abstract

Pneumothorax in patients with progressive systemic sclerosis (PSS) often presents as a difficult-to-treat disease. Autologous blood-patch pleurodesis has previously been used for the treatment of pneumothorax. Blood outside its own environment is an irritant; therefore, chest physicians must watch closely for an allergic reaction. The injection is simple, painless, causes no side effects, is an inexpensive treatment for pneumothorax and is available not only in patients with persistent air leak but also in those with residual air space. A case is reported here of blood-patch pleurodesis for pneumothorax in lung fibrosis due to PSS. As an alternative therapy for difficult-to-treat pneumothorax in patients with PSS with persistent air leak and residual air space, autologous blood-patch pleurodesis would be one of the treatment options.

## Introduction

Pneumothorax is not a rare complication of lung fibrosis due to progressive systemic sclerosis (PSS); however, a number of patients with PSS who have extensive pulmonary fibrosis with enlarged sub-pleural blebs and honeycomb lungs have been shown to develop pneumothorax. Various treatment options have been described (1). Surgery is a treatment option for recurrent and refractory pneumothorax. Chemical pleurodesis with talc or minocycline is also another treatment option, but there were complication with deterioration of pulmonary fibrosis especially in patients with pulmonary fibrosis. However, the success rate of the of PSS patients with pneumothorax appeared to be unsatisfactory. A case is reported here following successful treatment with a blood patch introduced into the chest via an intercostal chest drain. The purpose of this case report is to report the usefulness of this successful treatment.

## Case report

**Figure 1:**
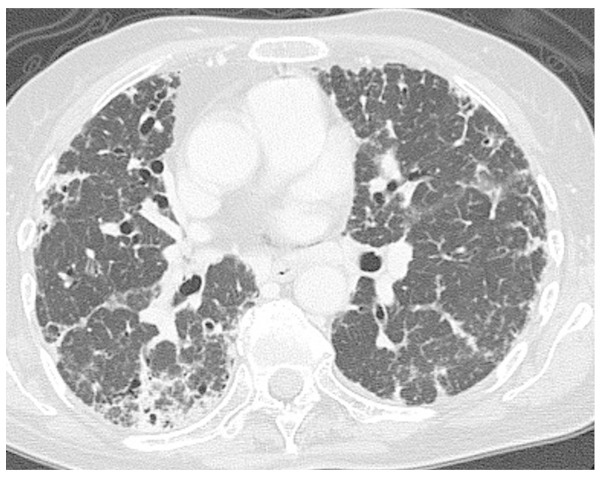
Chest computed tomography scan showing lung fibrosis at the time of diagnosis of progressive systemic sclerosis.

A 69-year-old female was admitted to the Mito Medical Center (Mito, Japan) due to dyspnea on exertion and right chest pain lasting all day. Three years previously she was diagnosed as having PSS with lung fibrosis (Fig. 1). Since then, the patient had experienced a pneumothorax on the right side several times and had subsequently received a video-assisted thoracoscopic surgery for persistent pneumothorax.

**Figure 2:**
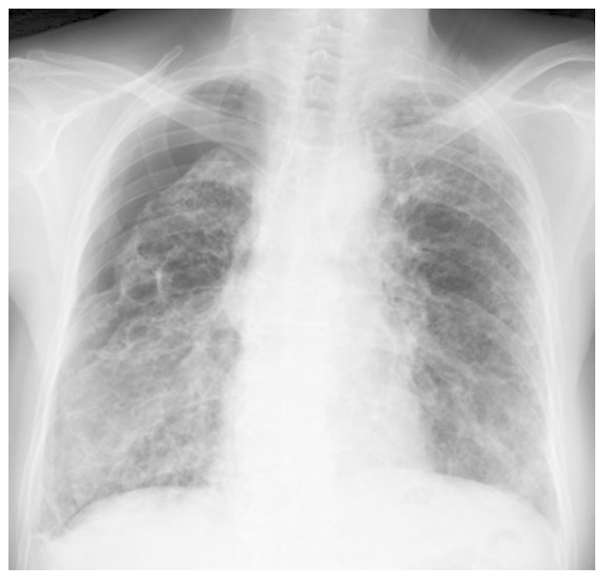
Chest radiograph reveals right lung pneumothorax, and reticulonodular opacities predominant in the bilateral lower lungs.

**Figure 3:**
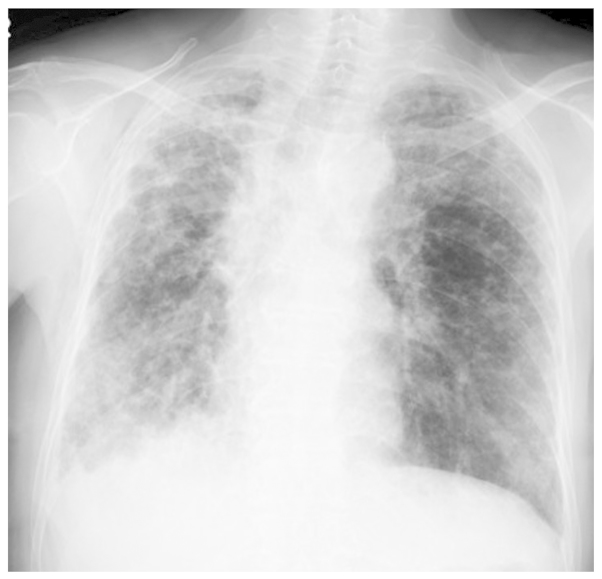
Chest radiograph 8 months after the autologous blood-patch pleurodesis shows no recurrence of pneumothorax.

Physical examination on admission revealed decreased respiratory sound in the right lung and diffuse fine crackles in both lungs. Skin thickening and tightness were observed on the fingers of both hands. Laboratory data on admission were as follows: White blood cell, 4,900/μl; C-reactive protein, 3.77 mg/dl; anti-nuclear antibody, 1:640; anti-centromere antibody, 1:640; rheumatoid factor, 4 U/ml. Tests for anti-neutrophil cytoplasmic antibodies to proteinase 3 and myeloperoxidase and anti-ribonucleoprotein and -topoisomerase 1 antibodies were negative. A chest radiograph revealed pneumothorax in the right lung, and reticulonodular opacities predominant in the bilateral lower lungs (Fig. 2). A 20 French intercostal chest tube (Argyle thoracic catheter, Covidien, Tokyo, Japan) was inserted into the pleural cavity, but the air leak continued and no improvement of the pneumothorax was obtained. The patient declined surgery; therefore, considering the deterioration of her respiratory condition following pleurodesis by other chemical agents, pleurodesis through the instillation of an autologous blood-patch was selected. A total of 50 ml autologous blood was injected via the chest tube. The tube was clamped for 3 h and connected to suction. The procedure was repeated twice over the next two days. Discontinuation of the air leak was achieved in three days and the chest tube was removed. Eight months later the patient was still well and attending the outpatient clinic without any recurrent pneumothorax (Fig. 3). This therapy was approved by the National Health Insurance of Japan as a postoperative persistent air leak therapy and by the Ethics Committee of the Mito Medical Center, University of Tsukuba-Mito Kyodo General Hospital (Mito, Japan). Informed consent was obtained from the patient.

## Discussion

PSS is a systemic disease that sometimes affects the lungs, resulting in lung fibrosis (1). Diffuse interstitial fibrosis is the most common pulmonary manifestation and pneumothorax is usually associated with the pulmonary complication of interstitial fibrosis (1). The underlying mechanism of pneumothorax is believed to result from the rupture of acquired subpleural cystic spaces associated with the diffuse interstitial fibrosis (2). In patients with PSS with interstitial fibrosis, the distinctive rigidity of lung parenchyma may prevent pre-expansion of the ruptured lung. Additionally, immunosuppressants including corticosteroids, which are frequently used for PSS, may aggravate persistent air leak; therefore, pneumothorax in patients with PSS often presents as a difficult-to-treat disease and prognosis is predicted to be poor. Chemical pleurodesis with tetracycline or talc has been successfully used (3–5), but pleurodesis is more usually performed once the air leak has resolved. Chemical pleurodesis using such agents can contribute to the onset of acute exacerbation of the interstitial fibrosis (4). Furthermore, if pleurodesis can be successfully achieved, the development of constrictive respiratory impairment may occur due to pleural thickening as a result of the chemical pleurodesis. If there is no improvement despite conservative treatment, a more invasive approach may be necessary. Video-assisted thoracic surgery is the next option for patients with recurrent pneumothorax and those for whom conservative treatment was not successful (5,6). Due to the severity of their underlying disease itself and their respiratory condition, these patients are often not suitable candidates for surgical treatment. As a consequence, their optimal management may be eventful. As an alternative therapy, autologous blood-patch pleurodesis has been used for the treatment of pneumothorax (7–9). Blood outside its own environment is an irritant; therefore, chest physicians must watch closely for an allergic reaction. The injection is simple, painless, causes no side effects and is an inexpensive treatment for pneumothorax available not only for patients with persistent air leak but also those with residual air space (10). In the case reported here, recurrent pneumothorax developed shortly after surgical therapy for the disease and there was persistent air leak as well as residual air space. Due to the high risk of deterioration of the respiratory condition of the patient by tight chemical pleurodesis, we selected to seal the pleural space with the injection of autologous blood as a successful pleurodesis agent in the treatment of recurrent pneumothorax.

In conclusion, as an alternative therapy for difficult-to-treat pneumothorax in patients with PSS with persistent air leak and residual air space, autologous blood-patch pleurodesis would be one of the treatment options.
